# Ectopic axillary breast cancer in a male patient

**DOI:** 10.1002/ccr3.3336

**Published:** 2020-09-18

**Authors:** Nils Peil, Zsuzsanna Varga, Marjam J. Barysch, Cornelia Brüssow, Konstantin J. Dedes

**Affiliations:** ^1^ Breast Cancer Center Comprehensive Cancer Center Zürich University Hospital of Zurich Zurich Switzerland; ^2^ Department of Gynecology University Hospital of Zurich Zurich Switzerland; ^3^ Institute of Pathology and Molecular Pathology University Hospital Zurich Zurich Switzerland; ^4^ Department of Dermatology University Hospital Zurich Zurich Switzerland; ^5^ Department of Oncology University Hospital Zurich Zurich Switzerland

**Keywords:** axillary cancer, axillary tumor, breast cancer, ectopic breast tissue

## Abstract

Ectopic breast tissue can persist in the axilla due to lack of involution of mammary glands along the mammary lines. It is rare in men, and the malignant transformation to breast cancer has occasionally been described. Differential diagnosis of any axillary tumor should include breast cancer arising at ectopic sites.

## Graphical abstract

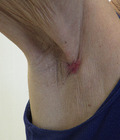

A 81‐year‐old male patient presented with an erythematous hard nodule on the right axilla (Figure 1). After excisional biopsy, histopathological findings showed invasive adenocarcinoma with apocrine characteristics. Histopathological criteria for a breast carcinoma of the NST type with apocrine differentiation were fulfilled. The tumor showed an infiltrative growth (Figure 2B), and the carcinoma cells consisted of eosinophilic cytoplasm, high‐grade nuclei, and prominent nucleoli (Figure 2A). The carcinoma cells expressed breast‐specific markers as Ck7, mammaglobin, NY‐BR‐1, brst2, and GATA3. The carcinoma was triple negative with Ki67 at 15‐20%.

**Figure 1:**
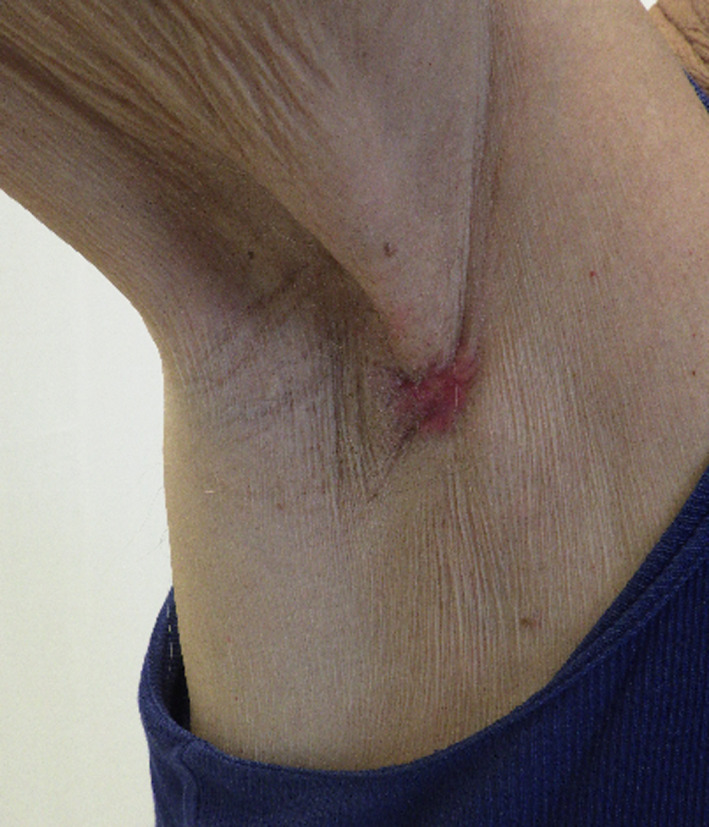
Preoperative picture of the lesion in the right axillary region

**Figure 2:**
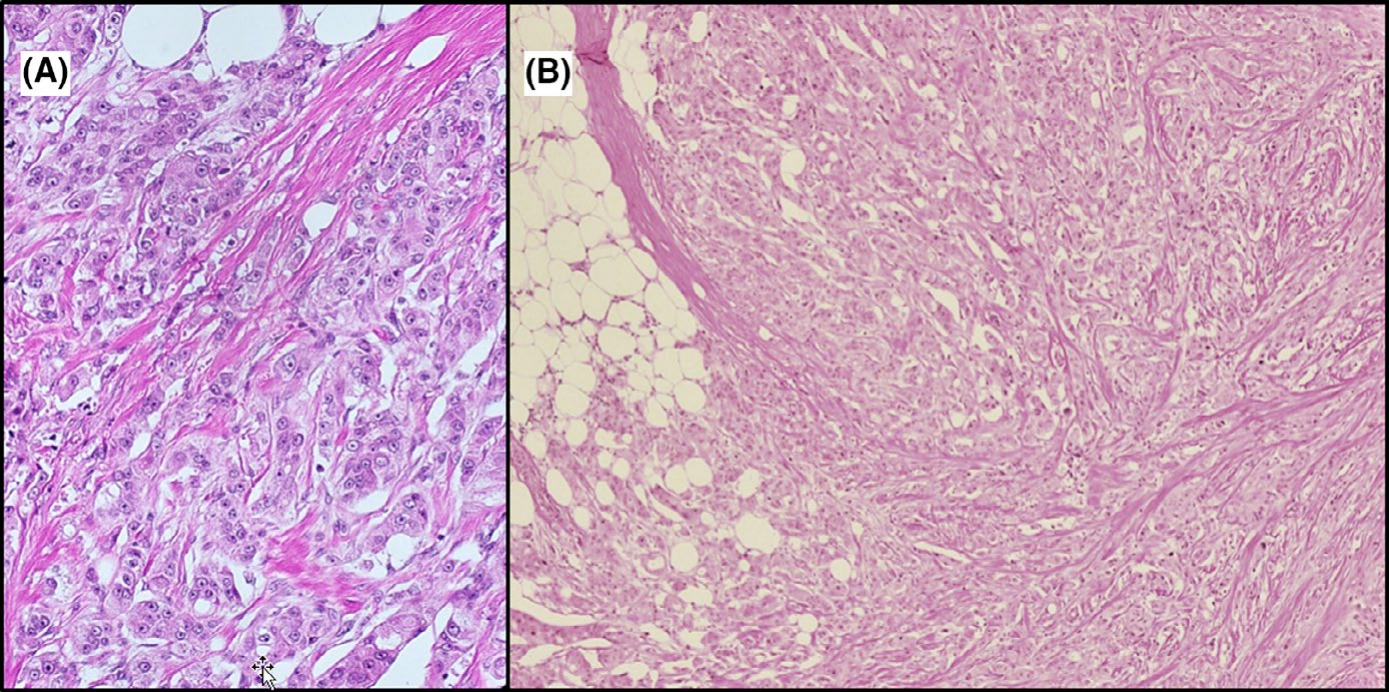
A, High power view of the apocrine type invasive breast carcinoma. The tumor consists of bright eosinophilic cytoplasm high‐grade nuclei and prominent nucleolus. B, In low power view, an overtly infiltrative growth is apparent. Hematoxylin‐eosin stain.

The histological features were consistent with a primary breast carcinoma in the axilla. Although there were no breast‐specific anatomical structures around the carcinoma, the whole histological and immunophenotypical context is diagnostic for primary breast carcinoma.

The patient underwent complete resection of the primary tumor, axillary lymph node dissection, followed by adjuvant regional radiotherapy.

## Conflict of Interest

All authors declare no conflict of interest related to this clinical image.

## AUTHOR CONTRIBUTIONS

Nils Peil: wrote the manuscript; Zsuzsanna Varga: analyzed and validated the histopathological findings; Marjam J. Barysch and Cornelia Brüssow: contributed to writing and reviewing manuscript; Konstantin J. Dedes: supervised the development of the manuscript.

## CONSENT STATEMENT

Published with written consent of the patient.

## Ethical approval

No ethical approval was necessary for this clinical image.

